# Synbiotic Supplementation Improves Obesity Index and Metabolic Biomarkers in Thai Obese Adults: A Randomized Clinical Trial

**DOI:** 10.3390/foods10071580

**Published:** 2021-07-07

**Authors:** Chaiyavat Chaiyasut, Bhagavathi Sundaram Sivamaruthi, Periyanaina Kesika, Suchanat Khongtan, Nanticha Khampithum, Subramanian Thangaleela, Sartjin Peerajan, Akkarach Bumrungpert, Khontaros Chaiyasut, Sasithorn Sirilun, Phakkharawat Sittiprapaporn

**Affiliations:** 1 Innovation Center for Holistic Health, Nutraceuticals, and Cosmeceuticals, Faculty of Pharmacy, Chiang Mai University, Chiang Mai 50200, Thailand; chaiyavat@gmail.com (C.C.); p.kesika@gmail.com (P.K.); suchanat_k@cmu.ac.th (S.K.); nanticha_khampithum@cmu.ac.th (N.K.); leelasubramanian@gmail.com (S.T.); sasithorn.s@cmu.ac.th (S.S.); 2 Health Innovation Institute, Chiangmai 50200, Thailand; s.peerajan@gmail.com; 3 Mahidol Nutrition Society, Faculty of Public Health, Mahidol University, Bangkok 10400, Thailand; abnutrition@yahoo.com; 4 Research Center of Nutraceuticals and Natural Products for Health & Anti-Aging, College of Integrative Medicine, Dhurakij Pundit University, Bangkok 10210, Thailand; 5 Institute of Research and Development, Chiang Mai Rajabhat University, Chiangmai 50300, Thailand; khontaros_cha@cmru.ac.th; 6 Neuropsychological Research Laboratory, Department of Anti-Aging and Regenerative Science, School of Anti-Aging and Regenerative Medicine, Mae Fah Luang University, Bangkok 11120, Thailand

**Keywords:** obesity, synbiotics, Lactobacillus, Bifidobacterium, inulin, fructooligosaccharide

## Abstract

The cluster of metabolic disorders includes obesity, dyslipidemia, hypertension, and glucose intolerance, increasing the risk of developing cardiovascular diseases and type 2 diabetes. Evolving proofs suggest an essential role of microbiota in human health and disease, including digestion, energy and glucose metabolism, immunomodulation, and brain function. The frequency of overweight is increasing, and the main causes for this are highly processed foods and less active lifestyles. Research is underway to unravel the probable relationship between obesity and intestinal microbiota. Here, we propose a method to understand and elucidate the synergistic function of prebiotics and probiotics in treating obesity. The biomarkers of obesity, such as cholesterol, gut permeability, oxidative stress, bacterial toxins, cytokines, and short-chain fatty acids, were analyzed in Thai obese individuals after being supplemented with a synbiotic preparation containing *Lactobacillus paracasei*, *Bifidobacterium longum*, *Bifidobacterium breve*, inulin, and fructooligosaccharide. The results reveal that the supplementation of synbiotics significantly altered the obesity-associated biomarkers in an appositive way. Further studies are warranted to use synbiotics as an adjuvant therapy for the management of obesity-related health issues.

## 1. Introduction

Obesity is one of the major health issues worldwide, leading to other health issues such as cardiovascular diseases, diabetes, and hypertension` which result in morbid obesity. A long-term imbalance in energy consumption, an irregular diet, altered gut microbiota, environmental factors, and genetic makeup are the primary causes of obesity [1]. According to a WHO report, about 650 million adults are obese, and 1.9 billion are overweight. Of these, possibly 38 million children (less than five years old) are obese [2].

The intestinal microbiota composition has a critical role in obesity [1]. For example, the Firmicutes to Bacteroidetes proportion was found to be higher in overweight/obese people compared to ordinary people. Energy absorption and storage may be associated with the balance of Firmicutes in intestinal microbiota [3,4]. The dysbiosis in intestinal microbiota is associated with cell homeostasis changes and affects the integrity of tight junctions, resulting in a decline in gut permeability [5]. Dysbiosis also influences inflammation, insulin resistance, and fat deposition, leading to the development of obesity. It increases the bacterial toxic load (i.e., lipopolysaccharide) in the host [6].

Probiotic bacteria are live microorganisms that confer a health benefit on the host when administered in suitable amounts. Recent studies have highlighted the beneficial effects of probiotics supplementation in hosts with metabolic disorders, cognitive declines, and cancers via the positive regulation of gut microbiota [1,7,8,9]. The supplementation of synbiotics (a mixture of probiotics and prebiotics) may effectively improve intestinal microbiota composition compared to probiotics or prebiotics supplements [10].

The combination of *Lactobacillus* and *Bifidobacterium*, along with prebiotics, could provide synergic effects to the host. So far, studies on the influence of the supplementation of synbiotic preparations containing *Lactobacillus paracasei*, *Bifidobacterium longum*, *Bifidobacterium breve*, inulin, and fructooligosaccharide on cholesterol profiles, cytokines, markers of leaky gut, antioxidant levels, and short-chain fatty acids (SCFAs) contents in Thai obese adults have not yet been reported. Thus, we aimed to study the effect of a synbiotic intervention on the biomarkers of cholesterol, gut permeability, oxidative stress, bacterial toxins, cytokines, and SCFAs in Thai obese subjects.

## 2. Materials and Methods

### 2.1. Study Design and Subjects

The study on the effects of synbiotics on obesity was conducted with randomized, double-blind placebo-controlled trials of Thai obese adults. The participants of this study provided their informed consent for participation before they joined the study. The Good Clinical Practices were followed in the study. The Ethics Committee of Mae Fah Luang University approved the study protocol (Code: REH-62151).

The inclusion criteria included Thai obese adults (BMI ≥ 25 kg/m^2^) according to the Asia-Pacific criteria, aged 18–65 years, who were willing to participate and complete the study. Subjects with kidney diseases, cardiovascular issues, gouty arthritis, and gastrointestinal tract discomforts were excluded from the study.

**Figure 1:**
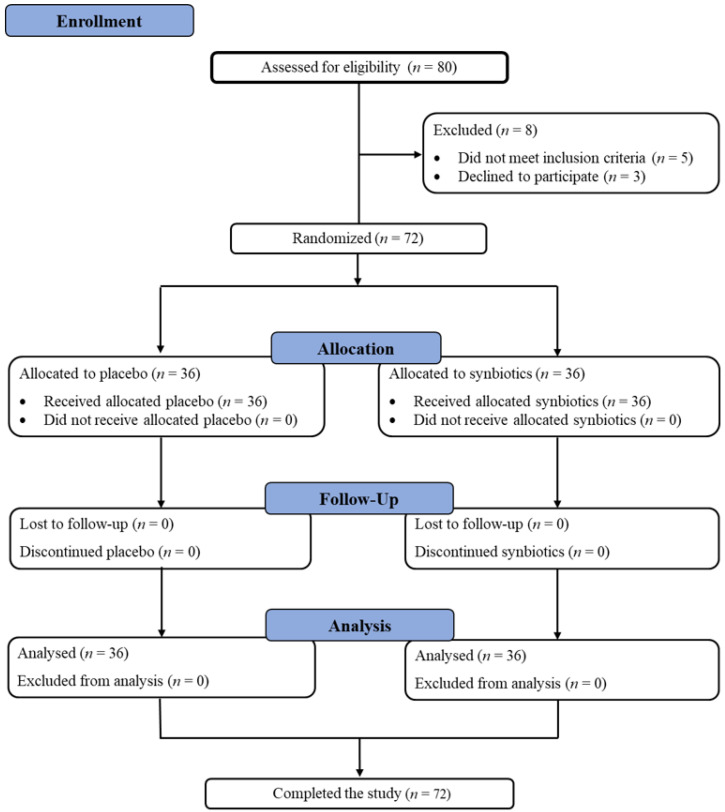
The study flowchart and enrollment.

Randomization was conducted with computer-generated codes using Random Allocation Software version 1.0.0 (Isfahan, Iran) [11]. The researchers and participants were blinded to the group assignment. Participants were randomized to receive either a synbiotic preparation (*Lactobacillus paracasei*, *Bifidobacterium longum*, *Bifidobacterium breve*, inulin, and fructooligosaccharide) or placebo for 12 week-long supplementations. After 12 weeks of supplementation, participants were asked to return for follow-up visits. The study flowchart and enrollment are described in Figure 1.

### 2.2. Treatment

Aluminum foil sachets containing 5 × 10^10^ CFU of probiotics (2 × 10^10^ CFU of *Lactobacillus paracasei*, 1 × 10^10^ CFU of *Bifidobacterium longum*, 2 × 10^10^ CFU of *Bifidobacterium breve*) and prebiotics (5 g of inulin and 5 g of fructooligosaccharide) were provided to the subjects in the synbiotic group. The concentration of *Bifidobacterium breve* was decided based on the anti-obesity effects of *B. breve* reported in a randomized, double-blind, placebo-controlled trial [12]. The combination of synbiotic and the concentration of other probiotics used in this study were based on our results (unpublished data). The probiotics were received from Lactomason Co., Ltd., (Gyeongsangnam-do, South Korea), and prebiotics were purchased from BENEO-Orafti S.A., (Oreye, Belgium). Those in the placebo group were provided with 10 g of corn starch. All subjects were instructed to regularly take the supplementation by dissolving the contents of one sachet in a glass of water before breakfast.

### 2.3. Assessments

#### 2.3.1. Clinical Data

The subjects’ personal history was assessed, including education, physical activities, smoking and alcohol drinking habits, and pharmacological treatments.

Demographic characteristics, including age, diabetes, alcohol drinking, and obesity index, were recorded manually. Body weight, body mass index (BMI), body fat, visceral fat, basal metabolic rate (BMR), and muscle were measured using an electronic scale (Picooc^®^, Model S1 Pro, Beijing, China).

#### 2.3.2. Laboratory Data

**Figure 2:**
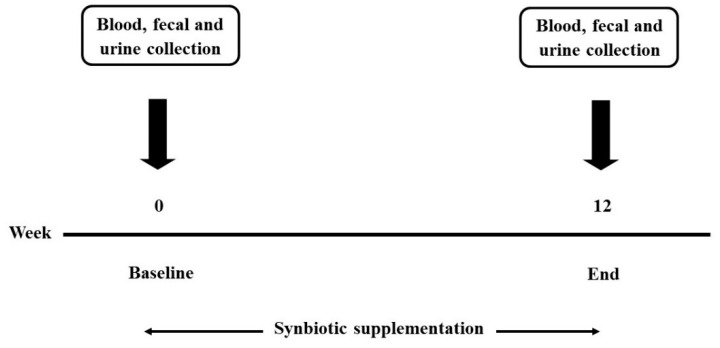
The timeline of this study.

Blood, fecal, and urine samples were collected at baseline and the end of the study (Figure 2). The biochemical analyses including total cholesterol (TC), HDL-cholesterol (HDL-C), LDL-cholesterol (LDL-C), triglycerides (TG), and fasting blood sugar (FBS) levels were determined from blood using the automated machine at AMS Clinical Service Center, Chiang Mai University, Chiang Mai, Thailand. Other biomarkers in the blood such as high sensitivity C-reactive protein (hs-CRP), immunoglobulin A (IgA), lipopolysaccharides (LPS), zonulin (ZO-1), and inflammatory chemokines/cytokines were determined using an ELISA commercial kit (OriGene Technologies, Rockville, MD, USA for hs-CRP, Elabscience^®^, Houston, TX, USA for IgA, MyBioSource^®^, San Diego, CA, USA for LPS and IDK^®^, Bensheim, Germany for ZO-1). Plasma total antioxidant capacity (TAC) was determined by a 2,2′-Azinobis (3-ethylbenzothiazoline-6-sulfonic acid) (ABTS) radical scavenging capacity assay [13,14]. The determination of malondialdehyde (MDA) was performed with the thiobarbituric acid reactive substances (TBARS) method [15,16]. The dismutation of superoxide radicals was determined using the assay of superoxide dismutase (SOD) [17], and reduced glutathione (GSH) in the plasma was determined using the recycling assay of 5,5′-dithiobis (2-nitrobenzoic acid) (DTNB) [18].

Fecal samples were collected to determine the short-chain fatty acids using high-performance liquid chromatography (HPLC) according to the following conditions: Shodex SH1011 as a column, 5 mM sulfuric acid as the mobile phase, with a flow rate of 0.6 mL/min at 210 nm and 75 °C [19,20], and putrefaction using HPLC with the following conditions: C18 (4.6 mm × 15 cm) as a column, methanol: water (60:40 *v*/*v*) as mobile phase, with a flow rate of 0.5 mL/min at 200 nm [21,22,23].

Urine samples were used to determine intestinal permeability. The subjects were given mannitol and lactulose at a ratio of 1:2, dissolved in water. After taking mannitol and lactulose, subjects were asked to collect urine within 6 h [24]. We measured the total urine volume from each subject and analyzed the intestinal permeability using a colorimetric commercial kit (EnzyChrom™, BioAssay, Hayward, CA, USA). Neuroinflammation markers in the urine, such as quinolinic acid (QA) and 5-hydroxyindoleacetic acid (5-HIAA), were determined using an ELISA commercial kit (Fivephoton Biochemicals™, San Diego, CA, USA for QA and Immusmol, Bordeaux, France for 5-HIAA).

#### 2.3.3. Statistical Analyses

Demographics were continuously analyzed using a t-test and discrete data using exact values. Data were analyzed using the paired t-test of means using STATA version 15.1 (StataCorp, College Station, TX, USA) for Windows licensed to the Faculty of Pharmacy, Chiang Mai University, Chiang Mai, Thailand. A descriptive analysis of the collected parameters was expressed as an absolute number and percentage. The continuous variables were represented as mean ± standard deviation (SD) or standard error of the mean (SEM) depending on their statistical distribution. The group’s data were calculated using a t-test and Gaussian regression analysis. The minimum level of statistical significance was set to *p* < 0.05 (two-tailed).

## 3. Results

A total of 72 subjects completed the study. There were no differences between synbiotic and placebo groups in terms of the initial measurements of age, body weight, BMI, body fat, visceral fat, muscle, arm, waist, and hip circumferences, waist/hip ratio, blood urea nitrogen content, creatinine, aspartate aminotransferase, and alanine aminotransferase, except in their BMR (Table 1).

There were no changes in all studied parameters after 12 weeks in the placebo group compared with baseline values. In the synbiotic group, significant differences were observed after 12 weeks of supplementation in body weight, BMI, body fat, waist circumference, waist/hip ratio, HDL-C, LDL-C, IL-6, IL-10, IL-1β, TNF-α, IgA, LPS, and ZO-1 values compared to the baseline values. No significant changes were observed in visceral fat, muscle, BMR, arm and hip circumferences, TC, TG, and hsCRP values in the synbiotic group (Table 2).

The antioxidant systems (TAC, MDA, GSH, total SOD, and Cu, Zn-SOD) of the subjects were documented. There were no statistically significant changes in the synbiotic and placebo groups after 12 weeks of supplementation (Table 3). The levels of butyric acid, propionic acid, acetic acid, and lactic acid were significantly changed after 12 weeks of synbiotic supplementation, whereas no changes were observed in the placebo group. The levels of lactulose, QA, the QA/5-HIAA ratio, cresol, and indole were significantly changed in the synbiotic group, which was not observed in the placebo group after 12 weeks (Table 3).

The significant changes in the studied parameters between the synbiotic and placebo groups after 12 weeks were calculated. The body weight, FBS, and cytokines, IgA, hsCRP, LPS, and QA levels were significantly altered compared to the placebo group (Table 4 and Table 5). There were no notable changes in the rest of the studied parameters between the synbiotic and placebo groups.

A Gaussian regression analysis of the data suggested that the synbiotic supplementation for 12 weeks significantly altered the body weight, body fat, muscle content, BMR, waist circumference, IL-6, IL-1β, TNF-α, LPS, ZO-1, lactulose/mannitol ratio, QA, 5-HIAA, QA/5-HIAA ratio, and butyric acid. There were no significant changes observed in cholesterol and antioxidant profiles (Table 6).

## 4. Discussion

The synergistic blend of both prebiotics and probiotics reduces plasma fasting insulin [25]. The most-used prebiotics are arabinoxylan and fructans [26]. The synbiotic supplementation of Bifidobacteria strains along with galactooligosaccharide may improve intestinal barrier function and possess anti-obesity effects [27].

There is a need for more approaches to aid in weight loss or to control obesity. Supplementation with *Lactobacillus plantarum* in obese mice reduced the deposition of adipose and upregulated the expression of lipid oxidative genes compared to control mice [28]. In order to treat obesity, *Lactobacillus* species can be used in combination with dietary management. *L. sakei* was found to impose anti-obesity effects when used in obese murine models [29,30]. The synbiotic supplements contained *L. acidophilus*, *Bifidobacterium lactis*, *B. longum* and *B*. *bifidum* as well as prebiotic galactooligosaccharide mixture, which increased the abundance of gut microbiome and also improved markers of metabolic syndrome as well as immune function in obese adults [31,32,33,34]. The supplementation of *L. gasseri* SBT2055-mediated fermented milk for 12 weeks reduced the weight and the abdominal visceral and subcutaneous fat mass in obese human subjects [35].

Treating obesity has been a long-term—but not well-defined—methodology that has been linked with gut microbial management. Even though there have been numerous research works carried out on obesity, the clarification needed regarding the treatment of obesity remains lacking. The present study was performed to inspect the impact of the supplementation of a synbiotics preparation containing *L. paracasei*, *B. longum*, *B. breve*, inulin, and fructooligosaccharide on body composition and metabolic biomarkers in Thai obese subjects.

The supplementation of pro-, pre-, and synbiotics to an organism might alter the secretion of some hormones and neurotransmitters as well as inflammatory factors that inhibit the avidity towards food, therefore reducing weight gain [36]. Many systemic reviews and meta-analyses provide evidence about synbiotics intake assisting the lipid profile and improving dyslipidemia [37]. Synbiotic supplements and foods potentially modulate the gut microbiota as well as improving the metabolism of lipids, insulin resistance, and liver enzymes to a greater extent than either pro- or prebiotics alone [38].

A well-known characteristic of probiotics is their involvement in an improved serum lipid profile through immunomodulatory properties [39]. They also may reduce inflammatory cytokines and Toll-like receptor 4 (TLR-4) activation, leading to a great impact on the serum lipid profile [40]. Probiotics integrate cholesterol in their cellular membrane [41] and convert it into coprostanol [42], resulting in a reduction in cholesterol absorption and serum total cholesterol levels by means of higher bile salt excretion [43,44]. It is a well-known fact that probiotics supplementation can modulate body weight and BMI if the tested individuals are treated for a longer duration. In addition to this, previous study suggests that the outcomes in weight reduction could be effective when prebiotics and probiotics are used together [45].

Overall findings from animal and human studies revealed the more beneficial functions of synbiotics in weight reduction and the modulation of the gut microbiome [27,46] compared to prebiotics and probiotics alone [47,48,49].

Obese individuals showed low-grade inflammation because of the increased production of cytokines, C-reactive proteins (CRP), interleukins (IL), tumor necrosis factor (TNF), and lipopolysaccharides (LPS) [50,51], which in turn resulted in metabolic dysfunction and obesity-linked disorders [52].

The dietary supplementation of synbiotics prepared using *L. gasseri* and galactomannan and inulin fibers reduced the weight and anti-inflammatory effects of synbiotic preparations along with *L. rhamnosus* (CGMCC 1.3724), *L. plantarum*, *L*. *paracasei* F19, *L. acidophilus* and LactisBb12, which together with oligo fructose and inulin showed beneficial effects on waist and hip circumference and BMI in obese people [53].

The randomized controlled trails in obese and prediabetes subjects showed variable results such as reduced TC, TG [31,54], and LDL levels [54,55], and the inflammation markers hs-CRP, TNF, LPS, and MDA were also found to be reduced [55,56,57]. Hotamisligil [58] and Lubberts [59] demonstrated that obese individuals express more TNF-α mRNA and protein when compared to lean controls. Thus, the increase in TNF-α induced IL-6 and IL-7 gene expression [60]. So far, the gathered evidence substantiates the role of peripheral 5-hydroxyindole-3-acetic acid (5-HIAA), the derivative end product of serotonin (5-HT) that is also involved in the pathogenesis of obesity and abnormal lipid and glucose metabolism [61]. In addition, 5-HIAA is associated with chronic low-grade inflammation, which in turn leads to metabolic syndrome. There is a strong association between serum 5-HIAA and central obesity [61]. However, 5-HT has long been known to be involved in the control of appetite, energy balance, and weight control [62,63]. Kinoshita and colleagues proved that 5-HT is responsible for adipocyte differentiation and might lead to adipogenesis and obesity [64]. Kim and colleagues showed that 5-HIAA is directly correlated with low-glyceride levels. Furthermore, there is a negative correlation between HDL cholesterol and 5-HIAA. In addition, an increase in 5-HIAA concentration increases plasma triglyceride levels, but the HDL cholesterol remains unaltered. Similarly, higher 5-HT concentrations were also detected in the blood of high-fat-diet-fed mice [65]. It is a well-known fact that zonulin is the physiological modulator of intestinal permeability and also a serum biomarker for impaired intestinal permeability [66,67,68]. The zonulin level was found to be elevated above the reference value in individuals with morbid obesity. S-zonulin was partially controlled after a 6-month-long conservative weight loss intervention and further reduced after bariatric surgery [69].

A meta-analysis by Ramezani Ahmadi and colleagues suggested that, compared to placebo, supplementation with pro/synbiotics pointedly reduced the serum zonulin level among selected subjects. Due to the comparison between probiotics and synbiotics, the finding of a significant level of serum zonulin reduction was only in subjects treated with probiotics [70]. The role of IL-1β in regulating adipose inflammation and fat-liver cross talk has been questioned. IL-1β regulates the lipid storage capacity in adipose tissues of the liver; however, in its absence, the adipose tissue expands, increasing in response to excess calories [71]. However, it is clear that IL-1β is a major promoter of adipose tissue inflammation in obese subjects [72].

Our results shows that 12 weeks of synbiotics supplementation significantly reduced body weight, BMI and body fat, visceral fat, BMR, and arm, waist, and hip circumferences compared to the placebo group (Table 1) in Thai obese subjects. The same parameters showed significant reductions in different time periods as well (Table 2). Reductions in IL-6, IL-1β, TNF-α, LPS, ZO-1, lactulose/mannitol ratio, QA, 5-HIAA, QA/5-HIAA ratio, and butyric acid levels were observed in the 12-week synbiotics-supplemented group (Table 6). The results support the notion that the potential use of synbiotics could be a promising choice for the treatment and/or management of obesity. This study may stimulate interest in molecular underpinnings beyond these significant results. Moreover, the study shows that synbiotic involvements in treating obesity could be a hopeful suggestive therapy in obesity and other related metabolic disorders.

## 5. Conclusions

The intake of synbiotics for a stipulated period of time had a moderating effect on body weight, BMI, body fat, visceral fat, BMR, and arm, waist, and hip circumference. The effects of synbiotic supplementation were proven to greatly reduce the above-mentioned parameters when administered for prolonged period of time. This evidence suggests that synbiotic supplementation produces a stronger effect compared to separate prebiotic and probiotic treatments. Additional anti-obesity effects can be obtained when obese subjects carry out synbiotic supplementation alongside any physical activity. The present study demonstrated that 12 weeks of synbiotic supplementation significantly reduced the physical parameters as well as the inflammation markers IL-6, IL-1β, TNF-α and other obesity markers including LPS, zonulin, 5-HIAA, and QA in Thai obese subjects. These obtained results offer a new platform to document other new markers and the effect of various other synbiotic supplementation combinations in the study of obesity.

## Figures and Tables

**Table 1:** Basic characteristics of the study subjects.

Parameters	Synbiotic Group	Placebo Group	*p-*Value
	(N = 36)	(N = 36)	
Age (years)	54.78 ± 1.92	58.94 ± 1.32	0.078
Body weight, cm	69.09 ± 1.90	68.17 ± 1.63	0.712
Body mass index, kg/m^2^	28.97 ± 0.77	30.01 ± 0.47	0.248
Body fat, %	33.09 ± 1.18	35.36 ± 0.87	0.125
Visceral fat, %	14.18 ± 0.88	15.36 ± 0.43	0.223
Muscle, %	56.48 ± 3.85	59.19 ± 1.44	0.497
BMR (kcal)	1409.42 ± 31.93	1323.04 ± 23.86	0.033 *
Arm circumference, cm	30.71 ± 0.49	30.62 ± 0.48	0.893
Waist circumference, cm	94.73 ± 1.92	95.79 ± 1.34	0.651
Hip circumference, cm	103.09 ± 1.38	104.33 ± 1.10	0.486
Waist/hip ratio	0.92 ± 0.01	0.92 ± 0.01	0.94
Diabetes, n (%)	7 (19.44%)	13 (36.11%)	0.188
Alcohol drinking, n (%)	6 (16.67%)	4 (11.11%)	0.735
Blood urea nitrogen (mg/dL)	14.89 ± 0.93	16.86 ± 1.84	0.699
Creatinine (mg/dL)	1.09 ± 0.09	1.08 ± 0.10	0.964
Aspartate aminotransferase (IU/L)	25.43 ± 4.62	24.73 ± 2.32	0.744
Alanine aminotransferase (IU/L)	27.59 ± 6.00	23.68 ± 2.97	0.925

* = Significant difference in *p*-value at 95% confidence interval. The proportion was analyzed using an exact probability test, and the continuous demographic data were analyzed using a *t*-test. BMR: basal metabolic rate.

**Table 2:** Changes in the studied parameters within groups at different times, expressed as mean ± SE.

Parameters	Synbiotic (N = 36)		*p-*Value	Placebo (N = 36)		*p-*Value
	Baseline	12 Weeks		Baseline	12 Weeks	
Body weight, cm	69.09 ± 1.90	67.45 ± 1.85	<0.001 *	68.17 ± 1.63	67.71 ± 1.71	0.067
Body mass index, kg/m^2^	28.97 ± 0.77	28.58 ± 0.75	0.017 *	30.01 ± 0.47	30.13 ± 0.58	0.662
Body fat, %	33.09 ± 1.18	31.96 ± 1.20	0.043 *	35.36 ± 0.87	36.27 ± 1.22	0.310
Visceral fat, %	14.18 ± 0.88	13.85 ± 0.79	0.162	15.36 ± 0.43	15.56 ± 0.52	0.445
Muscle, %	56.48 ± 3.85	56.08 ± 3.81	0.284	59.19 ± 1.44	59.01 ± 1.42	0.860
BMR (kcal)	1409.42 ± 31.93	1411.27 ± 29.61	0.898	1323.04 ± 23.86	1309.68 ± 24.87	0.102
Arm circumference, cm	30.71 ± 0.49	30.59 ± 0.57	0.808	30.62 ± 0.48	30.51 ± 0.53	0.795
Waist circumference, cm	94.73 ± 1.92	92.76 ± 1.84	0.009 *	95.79 ± 1.34	95.34 ± 1.45	0.648
Hip circumference, cm	103.09 ± 1.38	102.50 ± 1.30	0.419	104.33 ± 1.10	103.84 ± 1.31	0.705
Waist/hip ratio	0.92 ± 0.01	0.90 ± 0.01	0.018 *	0.92 ± 0.01	0.92 ± 0.01	0.961
Total cholesterol (mg/dL)	200.97 ± 8.40	195.50 ± 6.48	0.171	203.30 ± 8.11	199.97 ± 7.67	0.626
Triglyceride (mg/dL)	150.24 ± 16.04	145.97 ± 14.66	0.469	148.64 ± 11.04	149.88 ± 11.20	0.893
HDL-cholesterol (mg/dL)	50.21 ± 2.42	53.10 ± 2.53	0.030 *	50.42 ± 1.47	50.91 ± 2.56	0.813
LDL-cholesterol (mg/dL)	123.93 ± 8.61	112.66 ± 6.62	0.017 *	123.35 ± 7.35	116.48 ± 7.06	0.295
FBS (mg/dL)	111.79 ± 7.44	109.00 ± 6.02	0.373	109.68 ± 6.76	118.18 ± 6.89	0.084
IL-6 (pg/mL)	11.65 ± 1.17	7.24 ± 1.63	0.017 *	11.84 ± 0.49	11.82 ± 1.16	0.116
IL-10 (pg/mL)	1.04 ± 0.19	9.91 ± 2.04	0.018 *	1.56 ± 0.13	9.20 ± 5.00	0.153
IL-1β (pg/mL)	7.79 ± 0.76	5.42 ± 0.80	0.008 *	6.97 ± 0.64	6.29 ± 0.39	0.117
TNF-α (pg/mL)	13.75 ± 2.93	7.59 ± 1.54	0.011 *	9.25 ± 0.90	9.22 ± 0.56	0.679
IgA (ng/mL)	521.02 ± 69.33	636.48 ± 79.23	0.004 *	579.40 ± 54.02	504.73 ± 60.96	0.877
hsCRP (ml/L)	0.017 ± 0.006	0.008 ± 0.002	0.086	0.012 ± 0.001	0.015 ± 0.001	0.078
LPS (pg/mL)	108.99 ± 9.62	55.00 ± 6.09	<0.001 *	93.92 ± 7.87	81.42 ± 6.18	0.054
ZO-1 (ng/mL)	1.37 ± 0.17	0.98 ± 0.18	0.032 *	1.42 ± 0.17	1.41 ± 0.16	0.551

* = Significant difference in *p*-value at 95% confidence interval. HDL = High-Density Lipoprotein; LDL = Low-Density Lipoprotein; FBS = Fasting Blood Sugar; IL = Interleukin; TNF-α = Tumor Necrosis Factor alpha; IgA = Immunoglobulin A; hsCRP = High Sensitivity C-Reactive Protein; LPS = Lipopolysaccharide; ZO = zonulin.

**Table 3:** Changes in the studied parameters within groups at different times, expressed as mean ± SE.

Parameters	Synbiotic (N = 36)		*p-*Value	Placebo (N = 36)		*p-*Value
	Baseline	12 Weeks		Baseline	12 Weeks	
Lactulose	0.16 ± 0.03	0.07 ± 0.02	<0.001 *	0.12 ± 0.03	0.08 ± 0.02	0.135
Lactulose/mannitol ratio	0.20 ± 0.06	0.09 ± 0.01	0.072	0.14 ± 0.02	0.12 ± 0.02	0.315
QA (ng/mL)	23.53 ± 2.42	13.75 ± 1.71	<0.001 *	22.44 ± 1.69	24.25 ± 1.46	0.375
5-HIAA (mg/L)	5.04 ± 1.12	9.61 ± 1.95	0.051	4.00 ± 0.66	4.95 ± 0.93	0.642
QA/5-HIAA Ratio	3.14 ± 1.60	1.04 ± 0.46	0.008 *	5.76 ± 2.23	4.71 ± 1.77	0.756
Cresol (umol/g sample)	0.24 ± 0.03	0.09 ± 0.05	0.017 *	0.31 ± 0.16	0.14 ± 0.05	0.225
Indole (umol/g sample)	0.06 ± 0.01	0.04 ± 0.00	0.035 *	0.11 ± 0.06	0.06 ± 0.02	0.18
Skatole (umol/g sample)	0.07 ± 0.03	0.04 ± 0.00	0.285	0.05 ± 0.03	0.15 ± 0.07	0.285
Butyric acid (mmol/g sample)	38.01 ± 8.59	93.80 ± 18.96	0.002 *	46.40 ± 12.29	80.03 ± 32.27	0.311
Propionic acid (mmol/g sample)	259.16 ± 38.67	624.12 ± 82.82	<0.001 *	209.44 ± 72.32	466.52 ± 178.52	0.124
Acetic acid (mmol/g sample)	202.63 ± 37.70	425.89 ± 50.86	<0.001 *	206.56 ± 61.60	400.27 ± 69.40	0.161
Lactic acid (mmol/g sample)	54.42 ± 17.98	175.81 ± 36.88	0.002 *	92.11 ± 53.12	140.03 ± 57.00	0.866
TAC (µmol/mL)	0.195 ± 0.003	0.200 ± 0.011	0.664	0.180 ± 0.012	0.193 ± 0.008	0.08
MDA (µmol/mL)	0.45 ± 0.06	0.53 ± 0.09	0.301	0.52 ± 0.04	0.45 ± 0.03	0.157
GSH (µg/mL)	44.99 ± 17.46	30.98 ± 10.63	0.075	24.87 ± 6.86	17.29 ± 8.83	0.08
Total SOD (Units/mL enzyme)	56.91 ± 5.52	57.30 ± 6.28	0.854	54.01 ± 11.78	59.29 ± 7.24	0.492
Cu,Zn-SOD (Units/mL enzyme)	16.93 ± 3.13	44.57 ± 17.96	0.345	35.28 ± 7.34	39.49 ± 19.28	0.686

* = Significant difference in *p*-value at 95% confidence interval. QA = Quinolinic acid; 5-HIAA = 5-Hydroxyindoleacetic acid; TAC = Total Antioxidant Capacity; MDA = Malondialdehyde; GSH = Glutathione Reduced; SOD = Superoxide Dismutase.

**Table 4:** Comparison of the changes in studied parameters between groups. Changes represent the difference between baseline and at the end of the study.

Parameters	Baseline–12 Weeks		*p-*Value
	Synbiotic (N = 36)	Placebo (N = 36)	
Body weight, cm	−1.64	−0.46	0.002 *
Body mass index, kg/m^2^	−0.39	0.13	0.128
Body fat, %	−1.13	0.92	0.068
Visceral fat, %	−0.32	0.19	0.242
Muscle, %	−0.40	−0.18	0.448
BMR (kcal)	1.85	−13.36	0.483
Arm circumference, cm	−0.12	−0.11	0.809
Waist circumference, cm	−1.97	−0.45	0.113
Hip circumference, cm	−0.59	−0.49	0.51
Waist/hip ratio	−0.014	0.001	0.604
Total cholesterol (mg/dL)	−5.47	−3.33	0.695
Triglyceride (mg/dL)	−4.28	1.24	0.521
HDL-cholesterol (mg/dL)	2.9	0.48	0.066
LDL-cholesterol (mg/dL)	−11.28	−6.87	0.599
FBS (mg/dL)	−2.79	8.5	0.043 *
IL-6 (pg/mL)	−4.41	−0.02	0.010 *
IL-10 (pg/mL)	8.87	7.64	0.142
IL-1β (pg/mL)	−2.37	−0.69	0.041 *
TNF-α (pg/mL)	−6.16	−0.04	0.005 *
IgA (ng/mL)	115.46	−74.67	0.049 *
hsCRP (ml/L)	−0.009	0.003	0.002 *
LPS (pg/mL)	−53.99	−12.50	0.002 *
ZO-1 (ng/mL)	−0.39	−0.01	0.061

* = Significant difference in *p*-value at 95% confidence interval. HDL = High-Density Lipoprotein; LDL = Low-Density Lipoprotein; FBS = Fasting Blood Sugar; IL = Interleukin; TNF-α = Tumor Necrosis Factor alpha; IgA = Immunoglobulin A; hsCRP = High Sensitivity C-Reactive Protein; LPS = Lipopolysaccharide; ZO = zonulin.

**Table 5:** Comparison of the changes in studied parameters between groups. Changes represent the difference between baseline and at the end of the study.

Parameters	Baseline–12 Weeks		*p-*Value
	Synbiotic (N = 36)	Placebo (N = 36)	
Lactulose	−0.08	−0.04	0.002 *
Lactulose/mannitol ratio	−0.11	−0.02	0.508
QA (ng/mL)	−9.78	1.8	<0.001 *
5-HIAA (mg/L)	4.58	0.94	0.157
QA/5-HIAA Ratio	−2.10	−1.05	0.095
Cresol (umol/g sample)	−0.15	−0.16	0.661
Indole (umol/g sample)	−0.03	−0.05	0.379
Skatole (umol/g sample)	−0.031	0.103	0.121
Butyric acid (mmol/g sample)	55.79	33.64	0.229
Propionic acid (mmol/g sample)	364.96	257.09	0.258
Acetic acid (mmol/g sample)	223.25	193.71	0.47
Lactic acid (mmol/g sample)	121.39	47.92	0.162
TAC (µmol/mL)	0.005	0.013	0.557
MDA (µmol/mL)	0.08	−0.07	0.117
GSH (µg/mL)	−14.01	−7.59	0.584
Total SOD (Units/mL enzyme)	0.39	5.28	0.917
Cu,Zn-SOD (Units/mL enzyme)	27.64	4.21	0.251

* = Significant difference in *p*-value at 95% confidence interval. QA = Quinolinic acid; 5-HIAA = 5-Hydroxyindoleacetic acid; TAC = Total Antioxidant Capacity; MDA = Malondialdehyde; GSH = Glutathione Reduced; SOD = Superoxide Dismutase.

**Table 6:** Gaussian regression analysis summary at week 12 of supplementation for synbiotic group.

Parameter	Coefficient	95% CI	*p-*Value
Body weight, cm	−1.76	(−3.17 to −0.34)	0.018 *
Body mass index, kg/m^2^	0.123	(−0.64 to 0.88)	0.744
Body fat, %	−2.55	(−4.74 to −0.37)	0.023 *
Visceral fat, %	−0.17	(−0.96 to 0.61)	0.651
Muscle, %	−5.13	(−8.82 to −1.44)	0.027 *
BMR (kcal)	57.27	(2.77 to 111.76)	0.040 *
Arm circumference, cm	−0.14	(−2.61 to 2.33)	0.909
Waist circumference, cm	−2.73	(−5.23 to −0.23)	0.033 *
Hip circumference, cm	−4.54	(−10.06 to 0.97)	0.103
Waist/hip ratio	−0.02	(−0.05 to 0.01)	0.131
Total cholesterol (mg/dL)	−8.01	(−26.91 to 10.90)	0.397
Triglyceride (mg/dL)	−0.02	(−23.02 to 22.97)	0.998
HDL-cholesterol (mg/dL)	3.22	(−1.84 to 8.27)	0.207
LDL-cholesterol (mg/dL)	−10.57	(−26.42 to 5.28)	0.186
FBS (mg/dL)	−2.24	(−16.42 to 11.94)	0.751
IL-6 (pg/mL)	−4.50	(−8.78 to −0.23)	0.040 *
IL-10 (pg/mL)	5.18	(−9.96 to 20.32)	0.477
IL-1β (pg/mL)	−1.43	(−2.78 to −0.08)	0.039 *
TNF-α (pg/mL)	−4.26	(−6.51 to −2.01)	0.001 *
IgA (ng/mL)	117.99	(−55.97 to 291.95)	0.179
hsCRP (ml/L)	−0.003	(−0.011 to 0.005)	0.497
LPS (pg/mL)	−32.59	(−53.68 to −11.49)	0.004 *
ZO-1 (ng/mL)	−0.57	(−1.08 to −0.06)	0.032 *
Lactulose	−0.02	(−0.07 to 0.02)	0.319
Lactulose/mannitol ratio	−0.12	(−0.20 to −0.04)	0.008 *
QA (ng/mL)	−8.22	(−16.04 to −0.40)	0.041 *
5-HIAA (mg/L)	8.59	(0.68 to 16.50)	0.036 *
QA/5-HIAA Ratio	−7.15	(−13.69 to −0.61)	0.035 *
Cresol (umol/g sample)	0.09	(−0.30 to 0.48)	0.583
Indole (umol/g sample)	−0.004	(−0.054 to 0.046)	0.865
Skatole (umol/g sample)	−0.47	(−2.07 to 1.12)	0.165
Butyric acid (mmol/g sample)	59.74	(20.30 to 99.17)	0.009 *
Propionic acid (mmol/g sample)	−171.28	(−541.06 to 198.51)	0.335
Acetic acid (mmol/g sample)	−111.03	(−324.73 to 102.68)	0.28
Lactic acid (mmol/g sample)	6.73	(−135.27 to 148.73)	0.919
TAC (µmol/mL)	−0.03	(−0.09 to 0.04)	0.284
MDA (µmol/mL)	0.43	(−0.36 to 1.22)	0.18
GSH (µg/mL)	7.48	(−6.37 to 21.33)	0.208
Total SOD (Units/mL enzyme)	−32.33	(−91.18 to 26.52)	0.179
Cu,Zn-SOD (Units/mL enzyme)	−74.39	(−203.46 to 54.67)	0.131

* = Significantly difference in *p*-value at 95% confidence interval. Compare with the placebo group at week 12, HDL = High-Density Lipoprotein; LDL = Low-Density Lipoprotein; FBS = Fasting Blood Sugar; IL = Interleukin; TNF-α = Tumor Necrosis Factor alpha; IgA = Immunoglobulin A; hsCRP = High Sensitivity C-Reactive Protein; LPS = Lipopolysaccharide; ZO = zonulin; QA = Quinolinic acid; 5-HIAA = 5-Hydroxyindoleacetic acid; TAC = Total Antioxidant Capacity; MDA = Malondialdehyde; GSH = Glutathione Reduced; SOD = Superoxide Dismutase.

## Data Availability

The data presented in the manuscript is available on request from the corresponding author.
